# An ADePT evaluation for incorporating the TIPPS periodontal health intervention into primary care antenatal programmes to enhance infant birth weight in Palestine: a feasibility study

**DOI:** 10.1186/s40814-021-00827-x

**Published:** 2021-04-01

**Authors:** Lamis Abuhaloob, Nahla Helles, Peter Mossey, Ruth Freeman

**Affiliations:** 1 Dental School and Hospital, University of Dundee, Park Place, Dundee, Scotland DD1 4HN UK; 2 Ministry of Health, Gaza Strip and West Bank, Palestine

**Keywords:** Maternal periodontal health, Oral health education, Infant birth weight, Feasibility study, Palestine

## Abstract

**Background:**

A feasibility study was conducted to implement the *T*alk, *I*nstruct, *P*ractice, *P*lan and *S*upport (TIPPS) intervention for pregnant women to enhance infant birth weight in a conflict area in *L*ow- and *M*iddle-*I*ncome *C*ountries (LMIC). The decision tool, *A* process for *De*cision-making after *P*ilot and feasibility *T*rials (ADePT), examines the methodological factors identified in a feasibility study, that may require modification for a full trial. Thus, this study aimed to use the ADePT decision tool to evaluate if the feasibility study had achieved its objectives and to identify the need for intervention, clinical context and trial design modification.

**Methods:**

A one-arm, pretest–posttest feasibility study recruited 25 pregnant women in their first trimester and clinic staff from a primary healthcare clinic located in Gaza City, Palestine. The TIPPS periodontal health intervention was delivered by antenatal care nurses to the pregnant women during their regular follow-up appointments. The ADePT framework was applied to evaluate the findings from the feasibility study. The ADePT checklist demonstrated sample size estimation, recruitment, consent, intervention adherence, intervention acceptability, costs and duration, completion and appropriateness of outcome assessments, retention, logistics, and synergy between protocol components.

**Results:**

All recruited pregnant women (25, aged 16–35 years old) consented to participate in the study, and the adherence to the intervention was 88% (22 women). The TIPPS intervention was acceptable, but there was ambivalence over who should deliver it in the clinic. Only the cost of toothbrushing and TIPPS information materials was calculated, while the cost of nurses’ time was not included. The missing values of data were few (12% of gingival bleeding data and 22% from infant birth weight data). This intervention significantly reduced the mean percentage of plaque and bleeding scores after 3 months. The sample size for future randomised controlled trial was estimated around 400 participants. The participants stated the value of the intervention. The clinic staff voiced concerns regarding time and the cost of nurses providing the TIPPS intervention. This allowed suggestions to be made regarding the modification of trial design and context of implementation.

**Conclusions:**

The ADePT evaluation showed it was possible to progress to full trial with modifications in the trial design.

## Key messages regarding feasibility

- *What uncertainties existed regarding the feasibility?* The outcome of this study supported the feasibility of implementing TIPPs intervention into Primary Health Care in Palestinian Ministry of Health. No uncertainty was identified from this study except the effectiveness of TIPPs intervention on improving infant birth weight.
- *What are the key feasibility findings?* Twenty-two pregnant mothers (88%) completed the second follow-up appointment. This intervention significantly reduced the mean percentage of plaque and bleeding scores and was acceptable and valued by antenatal care staff and participants. The sample size for future randomised controlled trial was estimated around 400 participants. An ADePt evaluation allowed the identification of the appropriate modifications for future trial design.
- *What are the implications of the feasibility findings for the design of the main study?* The outcome measures of this feasibility study were satisfactory. The full trial should be multicentred. In addition, the staff raised concerns about staff time, cost of nurses’ time and expertise to deliver TIPPS; therefore, it would be recommended to employ community health workers to deliver TIPPS intervention and to integrate the economic evaluation into trial design.

## Introduction

During acute periods of political unrest and violent conflict, people’s access to routine secondary and specialist facilities drops, with a greater reliance on alternative forms of healthcare [1]. In response to unavailable and inaccessible healthcare in conflict areas, Spiegel and Checchi [2] called for a radical rethink of ‘health policies and mass interventions’. Central to their call was the incorporation of new healthcare services into existing ones and the ‘mass delivery of interventions for maternal and neonatal health’. They proposed that doing so would build capacity through ‘healthcare personnel, [*existing*] centres’ with a focus upon ‘primary and emergency healthcare services’.

Spiegel and Checchi [2] appeal for improved and accessible primary care in which new services use current infrastructure and in which healthcare personnel has some relevance for pregnant women residing in areas of violence and conflict. In such situations, women are four times at greater risk of having a low birth weight infant—a major cause of infant death [3–6]. For women in Gaza, poor periodontal health was shown, in addition, to have a significant and injurious effect on infant birth weight [7].

Anecdotal evidence [8] proposes that periodontal disease is common among pregnant women in areas of conflict, and the European Federation of Periodontology [9] and later the *Cochrane Review* further suggested that periodontal interventions to improve maternal gum health may enhance infant birth weight (when the intervention is delivered in the first trimester) [10]. Therefore, it seems reasonable to postulate that an evidence-based periodontal intervention to improve gum health could enhance infant birth weight for women residing in conflict zones.

*T*alk, *I*nstruct, *P*ractice, *P*lan and *S*upport (TIPPS) is a psychologically informed and evidence-based intervention to promote gum health [11], developed by Clarkson et al. [12] for primary care settings which may be delivered by appropriately trained healthcare personnel. It seemed that TIPPS could, therefore, be used to promote gingival health in pregnant women in the first trimester of pregnancy. Moreover, TIPPS, as a new service, could be merged into existing primary healthcare antenatal clinics and be delivered by appropriately trained healthcare personnel. However, a series of questions remained as to how feasible it would be to introduce a new preventive intervention into an existing primary healthcare scenario. For instance, how acceptable would it be to antenatal staff? Would pregnant women wish to participate? Would it be possible to gather a sample and retain participants for a follow-up? Therefore, a feasibility study was conducted to test the feasibility and acceptability of incorporating the TIPPS periodontal intervention into a primary healthcare antenatal clinic, for women in the first trimester of their pregnancy, delivered by appropriately trained nursing staff. The purpose of the feasibility study was (1) to implement the TIPPS intervention for pregnant women in their first trimester to promote maternal periodontal health (reduced gingival bleeding) and enhance infant birth weight (in kilograms) and (2) to conduct a process evaluation to assess the acceptability of the intervention to staff and pregnant women, the recruitment rate, participant retention, the willingness of nurses to recruit, nursing staff, willingness to be trained and provide TIPPS, quality of the collected data, appropriate sample size, and the suitability of the primary (infant birth weight) and secondary (gingival bleeding and plaque accumulation) outcome measures.

The decision tool known as ADePT: ‘*A* process for *De*cision-making after *P*ilot and feasibility *T*rials’ by Bugge and Williams [13] examines the ‘methodological issues’ identified in a feasibility study that may hinder or facilitate progression to a full trial. ADePT permits an examination of the evidence from the feasibility study and the degree to which ‘methodological issues’ such as recruitment, retention, and adherence and acceptability of the intervention, among others, have been successful. From the feasibility study findings, ADePT provides a framework to decide whether there should be an amendment of the intervention, the context of its delivery and a trial design before embarking on a full trial. The aim of the study was to use the ADePT decision tool to evaluate whether the feasibility study had achieved its objectives and to identify the need for intervention, clinical context and trial design modification.

Based on that, the objectives of this study were

1. To implement the TIPPS intervention for pregnant women in their first trimester
2. To conduct a process evaluation using ADePT tool to assess the acceptability of size estimation, recruitment, consent, intervention adherence, intervention acceptability, costs and duration, completion and appropriateness of outcome assessments, retention, logistics, and synergy between protocol components to proceed to full trial

## Methods

### Setting and context

The *Pan Arab Survey for Family Health*, conducted in 2004, has indicated that 95.5% of pregnant women received antenatal care [14]. The Maternal and Child Health Care Programme, Ministry of Health, Palestine includes antenatal, perinatal, and neonatal health care, including child vaccination programmes. A primary healthcare clinic supported by World Health Organisation (WHO) and Maternal and Child Health Care Department programmes, located in Gaza City, agreed to act as the centre for the feasibility study. The study was conducted with the support of the Maternal and Child Health Care Department, Ministry of Health, Palestine.

### Sample

A convenience sample of pregnant women in their first trimester of pregnancy, who attended the primary healthcare clinic located in Gaza City for antenatal care, were approached by nurses and invited to participate. The women were eligible to participate if they were in the first trimester of their pregnancy, dentate, were able to brush their teeth, were not suffering from periodontal diseases or diabetes, and had provided written and informed consent.

Clinical and administrative staff working in the maternal primary healthcare clinic and providing antenatal care were invited to take part in a process evaluation of the feasibility study. They were eligible to participate if they were healthcare professionals or administrative staff who had provided antenatal care and had given informed and written consent.

A recruitment of twenty-five women was considered enough to help in estimating the full trial’s sample size based on feasibility study outcome.

### Ethical considerations

Ethical approval was gained from the University of Dundee (UoD\SDEN\2019018) and from the Palestinian Research Council (PHRC/HC/496/19).

Pregnant women were provided with participant information sheets. They were given time to consider the participant information sheet, and their questions were answered by the clinic nurses. Women who agreed to participate were invited to attend a baseline appointment, where additional questions were answered by a researcher (LA), and a written and informed consent was obtained. The nursing staff were given a participant information sheet by LA and given time to consider their participation. After a week, they were asked to sign the consent if they had chosen to participate.

### Study design

This feasibility study adopted a one-arm, pretest–posttest design to examine the intervention effects. Three waves of data were collected at baseline, post-intervention time 1 (1 month from the baseline visit), and post-intervention time 2 (3 months from the baseline visit).

### The TIPPS intervention

The aim of TIPPS is to enable patients to remove plaque using effective toothbrushing and interdental cleaning. The TIPPS intervention is patient-centred and evidence-based. It is underpinned by Social Cognitive Theory, which has been demonstrated to be effective in behaviour change interventions in oral health [12, 15]. TIPPS promotes periodontal health through the removal of plaque by effective toothbrushing. The first element of TIPPS is the *T*alk and *I*nstruct phase where advice and a demonstration of effective toothbrush with fluoride toothpaste is provided; the second part is to enable patients to *P*ractise and *P*lan their toothbrushing activities; and finally to *S*upport and increase confidence to ‘remove plaque and plan, how and when’ to care for their teeth and gums [11] (Table 1).

**Table 1:** The TIPPS intervention

The TIPPS interaction is patient-centred, lasts for several minutes, with more time being taken for patients with greater need. The intervention format, the language used, and clinical communication are dependent upon the patients’ health literacy and understanding. The emphasis is on tailoring the intervention to the patient’s needs using appropriate demonstrations of plaque removal using models or watching the patient and providing advice. There are 5 TIPPS phases which are tailored to the needs of the patient at baseline and at subsequent follow-up visits. The different phases are	
1	Talk is the opening phase of TIPPS. Talk includes a discussion of the causes of periodontal disease and the importance of oral hygiene in its prevention. Integral to this phase is the use of visual aids to describe periodontal disease and the imparting of knowledge such as brushing teeth with fluoride toothpaste twice a day for 2 min, using of a small-headed toothbrush, and using interdental cleaning once a day.
2	Instruct is the second phase in which the patient is shown how to clean their teeth effectively, using the toothbrush, fluoride toothpaste, dental floss, and interdental cleaning brushes.
3	Practise allows the patient to clean their teeth in front of the clinician who provides and requests feedback from the patient, with concerns raised, addressed.
4	Plan adopts a patient-centred approach as the clinician negotiates and, with the patient, develops an action plan for effective plaque removal. This plan is reviewed and modified as the patient moves from preparation, through action to maintenance of their oral hygiene behaviours.
5	Support is the final phase of TIPPS, and it allows the clinician to support the patient during the early phase of behaviour change and consolidation of toothbrushing and interdental clean as daily routines.

The antenatal nurses attended a 1-day TIPPS workshop. They were provided with information about gum disease, the various elements of TIPPS and given the opportunity to practise toothbrushing and interdental cleaning, to role play and to goal set. They were given the TIPPS educational materials (translated and back translated into Arabic) and shown the TIPPS training video. Throughout the day, they were encouraged to ask questions to ensure that they understood the elements of TIPPS and that the training had fulfilled their needs. Working in this way ensured the fidelity of the implementation of the TIPPS intervention.

The pregnant women received the tailored TIPPS intervention, together with a personalised and negotiated oral health hygiene plan, including toothbrushes, fluoride toothpaste, dental floss and interdental brushes as an integral part of their antenatal appointment by the nurses. At each of the two subsequent follow-up visits, the women were given additional fluoride toothpastes, toothbrushes, interdental brushes and dental floss.

### Examination of periodontal health

Using disposable mask and gloves, disposable mirrors and WHO Basic Periodontal Examination probes, plaque scores were used as a measure of oral cleanliness and bleeding on probing for gingivitis. Scores of 1 for plaque present and 1 for gingival bleeding were noted for all four sites of the teeth present and examined [11]. Percentage scores for upper and lower teeth were calculated separately to give percentage total scores for plaque and bleeding separately. The percentage score was calculated by adding the scores for the number of tooth surfaces with plaque together, multiplying by 100, and dividing by the total number of teeth times 4 [11]. This adaption of O’Leary et al.’s [16] method allowed comparisons to be made between baseline and follow-up appointments for plaque and bleeding on probing. The percentage plaque scores were conveyed to the nurses to be used to assist the women to achieve lower plaque scores and to increase their confidence in maintaining their oral hygiene practices.

### Process evaluation

Following the Medical Research Council’s [17] advice on process evaluation, a qualitative exploration was conducted. The process evaluation investigated the acceptability of providing the TIPPS intervention in the context of a pre-existing primary healthcare facility by antenatal nurses, the willingness of women to participate, the willingness of nursing staff to be trained and to provide the TIPPS intervention as part of their routine antenatal care, and the adherence to the intervention.

Three months after baseline, and following completion of the data collection, a series of in-depth semi-interviews were conducted with five people from the clinic including participating nursing and clinic staff. Five women who had not attended all of the appointments were contacted and interviewed. Five women who had attended all three appointments were invited for interview. All the interviews were recorded and transcribed at a later time.

### Completion of the outcome assessments

The quality of the data was taken as an indication of the completeness of the outcome assessment. This was assessed using the ‘completeness’ dimension of the Data Quality Assessment Framework [18]. The completeness of the data was measured by the amount of missing data of the primary and outcome variables at baseline and Time 2. This process included identification for any errors and ensuring that the collected data was appropriate to assess the change on maternal periodontal health and infant birth weight as a result of applying TIPPs intervention.

### Data analysis

The data were entered onto the Statistical Package for the Social Sciences (SPSS) data sheet (V22.0) and transferred to Stata (V16.0). The quantitative data was subjected to descriptive statistics and non-parametric measures of significance for related samples [19]. The intention was to calculate the sample size from the initial data collected in this study to enable a future definitive trial to be powered sufficiently. The transcripts were analysed using content analysis with Nvivo software. The ADePT framework was applied by LA to evaluate the findings from the feasibility study. Then it was reviewed and agreed by the research team.

## Results

### Recruitment, consent, retention, and sample size

A convenience sample of twenty-five mothers who met the inclusion criteria were recruited over a 3-week period. All of the mothers were 3 months into their pregnancy. This represented 10% of all of the women who attended the clinic over the 3-month period. All the women provided informed and written consent. The women were aged between 16 and 35 years old. Of the 25 mothers, 14 attended the appointment at Time 1, and 22 attended at Time 2. This gave an overall retention rate between baseline and Time 1 of 56% and between baseline and Time 2 of 88%. Four members of the nursing and administrative staff fulfilled the eligibility criteria and were invited to take part. All provided informed and written consent.

The decision to proceed to the full trial depended on ADePT evaluation outcome (Table 2). It explored the evidence from the feasibility study and potential methodological issues of future full trial, as follows:

**Table 2:** ADePT checklist: summary of the evidence to support to progression to full trial

Methodological issues^1^	Findings	Evidence
1. Sample size calculation	Completed using the outcome variable of gingival bleeding	The expected difference in mean percentage gingival bleeding between intervention and control was 9 (SD intervention group 31: SD control group 32). The power calculation for these figures at 80% with a two-sided alpha at 5% indicates a total sample size of 400.
2. Eligibility	Achieved: 10% eligible of women for inclusion; all antenatal clinical and administrative staff eligible	In the first 2 weeks of the study, a total of 300 pregnant women attended the clinic of whom 10% met the inclusion criteria. 15 women were approached in week 1 and a further 10 in week 2. All women approached agreed to participate. Four clinical and administrative staff were approached and all agreed to participate.
3. Recruitment	Recruitment was successful with all participants agreeing to participate	The antenatal clinic staff were very enthusiastic as their previous experience with dental health services had been positive. They felt that that dental health service should be re-instated.
4. Consent	All participants provided informed and written consent	After being provided with the PIS, all participants, after a cooling-off period, agreed to participate in the TIPPS intervention and provided informed and written consent.
7. Adherence to the intervention	The adherence to the intervention was good.	TIPPS intervention appointments attended: baseline: *n* = 25; Time 1: *n* = 14 (56%); Time 2: 22 (88%). The TIPPS training day was attended by all of the nurses, and administrative staff attended the day’s training workshop; enjoyed learning about TIPPS and implementing it in accordance with the TIPPS intervention protocol.
8. Intervention acceptability	The TIPPS intervention was acceptable but ambivalence over who should deliver it in the clinic	All of the women and the clinic staff felt that the TIPPS intervention was an excellent idea which should be extended throughout pregnancy. The clinic staff felt that others should implement it and not nursing staff. Concerns about staff time and cost of nurses’ time and expertise to deliver TIPPS.
9. Intervention costs and duration	Intervention costs were calculated and duration time of the total intervention known.	Costs of toothbrushes, toothpastes, and interdental cleaning together with TIPPS information materials. The total length of time of the intervention including follow-up appointments was 3 months.
10. Completion of the outcome assessments	Outcome assessments were completed	Assessed by the quality of the data: only 12% of data were missing from the outcomes variable, gingival bleeding and 22% from infant birth weight
11. Appropriateness of the outcomes measured	The measures used to assess the primary and secondary outcomes assessed issues of concern	Change in plaque and bleeding scores were measured. Completed infant birth weight will be secured on the birth of the child.
12. Retention	Retention was good for mothers at baseline and at Time 2 but not Time 1; retention for clinic and administrative staff was good	See point 7: the conflict affected 3 participants attendance at Time 2.
13. Logistics of running a feasibility study in a LMIC in an area of conflict	The recruitment went well due to XX’s specialist knowledge and interactions with the clinic staff; difficulties in transporting dental supplies to Gaza city experienced	25 women, 2 antenatal nurses, and 2 administrative staff recruited. Dental supplies were delivered to the clinic following intervention with CDOs in Palestine and Israel.
14. Synergy between all components of the protocol	All elements of the study worked well together	The elements of the study flowed well from one part to the next. Knowledge obtained informative for a full trial.

^1^Checklist in identical order to the ADePT decision tool. The randomisation criteria (5 and 6) were omitted as this did not form part of the feasibility study

### Sample size calculation and eligibility and consent of participants

Table 2 provides the details of the sample size calculation which indicated a total sample size of 400 participants for a full trial, together with details of eligibility and consent to participate.

### Process evaluation: adherence and acceptability of the intervention

Interviews were conducted with five women who attended at baseline and Time 2 appointments but had not attended the Time 1 appointment. From the remaining 17 women who attended all three appointments, five women were randomly chosen and invited to take part (Table 2).

All of the women interviewed stated they enjoyed TIPPS, found the TIPPS intervention helpful, and liked the information and support on how to brush their teeth correctly. They were also very grateful for the supply of toothbrushes and fluoride toothpaste free of charge—as one woman commented, *‘It is so important that you gave us the toothpaste and toothbrush, this is why we can brush our teeth*’. Many of the women commented that they had never been shown how to brush their teeth before and a consequence of the TIPPS programme was not only to train them how to brush their teeth effectively, ‘*My teeth are crowded, the interdental brush really helps me to clean between them*’, but also enabled them to take the TIPPS message home to help children brush their teeth. As one woman stated, ‘*I’ve taught my sons how to brush their teeth now, after learning about it here’*.

When asked about additional thoughts on the programme, they stated that the TIPPS programme should be part of the antenatal programme throughout pregnancy and that dental treatment should also be part of the intervention, with a facility to remove calculus and restore teeth. For the women who had not attended the first follow-up appointment (Time 1), the reasons for non-attendance were associated with family constraints and difficulties experienced in travelling to the clinic. These women felt strongly that while they saw the benefit of the TIPPS intervention, they would only attend if the appointments were at the same time and part of their antenatal appointment.

Five people from the clinical and administrative staff agreed to be interviewed. The nurses agreed with the opinions voiced by the women that the TIPPS intervention in general was an excellent idea, and they enjoyed learning about TIPPS and having the opportunity to implement it with the pregnant women. They appreciated the need for goal setting and while time-consuming, some believed that it was important, *‘because the life style and culture of people, their ways of tooth brushing and background about oral health importance differ from one area to anothe*r’. Some nurses and staff also stated that they believed in the importance of the programme and felt that for the TIPPS intervention to be widely appreciated it should, *‘involve many different areas - this would make it more representative and results generalizable’*. Despite this, they felt that they were too busy to recruit women into the programme and to provide the TIPPS intervention in already understaffed and busy clinics. A commonly voiced concern was that they felt they were too ill-informed to answer any other dental questions other than about gum health, toothbrushing, and interdental cleaning. They felt that a dentist should be employed for that purpose. A dental surgery should be provided at the clinic where the TIPPS intervention would be implemented and dental treatment provided by a dental team.

Potential risks to the successful implementation of TIPPS included the lack of engagement of some pregnant women to attend any appointments, the potential costs of toothbrushes, toothpaste, and interdental cleaning being unaffordable for the women and the costs to the clinic; the increased healthcare personnel costs were mentioned by the nurses and others. The following quotes are illustrative:

‘*There are mothers who do not collaborate with the dentist or nurse, do not return for their appointments and would not follow the instructions for taking care of their teeth. I believe these mothers are in need of more intensive oral health education lessons.*’

‘*The cost should be considered. Is it going to be frequently available or is it going to be available only for* [high risk] *groups*? *The budget* [for the programme] *needs to be high for staff time and resources. . .* [the] *antenatal care programme is already free of charge and if you ask a mother to buy toothpaste and brush, she may not be able to afford the cost.’*

Thinking to the future, the head of antenatal care stated that she felt that the TIPPS programme could experience difficulties, especially when violence had resulted in a previous dental treatment service being removed from the clinic, ‘*after the increase in political instability this programme was stopped’*. Concerns were raised with regard to the disruption of an already busy clinic, especially if the pregnant woman failed to attend or arrived late or on another day for their appointment, and the additional effect of TIPPS on an already busy clinic. The division of healthcare into definite disciplines was also voiced, ‘*highly skilled midwives involved in oral health care and education to run a programme which previously had dentists responsible for this work*.’

### Intervention costs and duration

The total costs for the provision of toothbrushes, toothpastes, and interdental cleaning aids, together with the costs of the TIPPS information materials for the training day and for the participating pregnant women were known. No economic evaluation was undertaken.

### Completion of the outcome assessments: quality of the data

At baseline there was no missing data for the primary and secondary outcome variables. Twelve percent of the secondary outcome variables was missing at Time 2, and 22% of the primary outcome variable was missing when collected from the mothers on the delivery of their babies (Table 2).

### Appropriateness of the outcomes measured

Three of the women were prima gravida; one woman had had a miscarriage. For the remaining 21 mothers, they had between 2 to 7 pregnancies and had between 1 and 5 surviving children. For the 21 mothers, the mean birth weight of their last infant was 3.25 (SD 0.46) kg with a range of 2.5–4.0 kg. Twenty-two mothers were contacted following the birth of their babies. The mean birth weight of their new infants for women with more-than-one previous pregnancy was 3.12 (SD 0.30) kg with a range of 2.5–3.6 kg (Table 2). Of the remaining three women who were prima gravida, two were contactable. One woman had twins, one of whom died and the other born prematurely at 0.75 kg (now a healthy baby), and the mean weight of the infant of the other first-time mother was 2.50 kg.

**Fig. 1:**
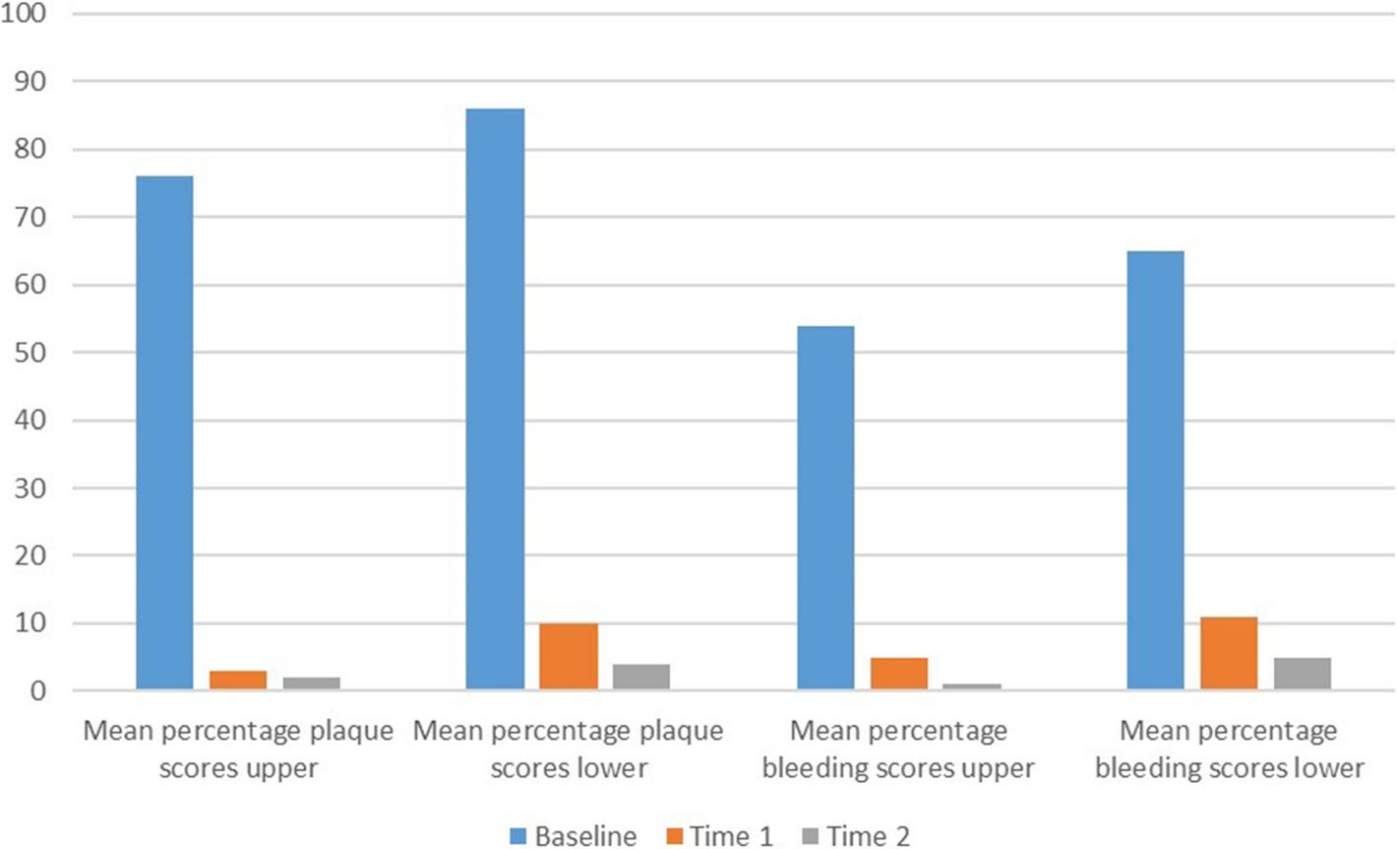
Changes in mean percentage plaque and bleeding scores over time for upper and lower teeth

Figure 1 shows the changes in mean percentage plaque and bleeding scores over time. There were statistically significant differences in mean rank percentage plaque scores from baseline to Time 2, for the upper teeth (*X*^2^[2] =21.51; *P* < 0.001) and lower teeth (*X*^2^[2] = 18.31; *P* < 0.001) across the three time points. There were also statistically significant differences in mean rank percentage bleeding scores from baseline to Time 2, for upper teeth (*X*^2^[2] = 25.24; *P* < 0.001) and lower teeth (*X*^2^[2] = 22.91; *P* < 0.001) across the three time points.

### Logistics of running the feasibility study in LMIC in an area of conflict

Recruitment of the pregnant women and clinic staff went well due to LA’s specialist knowledge, interactions with clinic staff, and support from the head of the clinic. However, difficulties transporting dental supplies from the UK to Gaza city were experienced. The toothbrushes, toothpastes, and examination kits were only able to be released from customs (Table 2) following the intervention of the Chief Dental Officers from Palestine and Israel.

### Synergy between all components of the protocol

The ADePT evaluation (Table 2) demonstrated that all of the elements of the study worked well together. The findings of the study increased our knowledge and the need for a dedicated place for the TIPPS intervention, where women would be happy to discuss their oral health problems and have trained oral health staff to provide the TIPPS intervention.

## Discussion

Inequality in oral healthcare is a growing global health issue that demands immediate action. In addition, the UN Sustainable Development Goals (SDGs), SDG3, SDG5, and SDG10 (better health and wellbeing, gender equality, and reducing inequalities respectively) and universal health coverage (UHC) have emphasized on the importance to proceed to action, commencing with research to improve our understanding of what actions and policies would be appropriate. This research needs to be feasible and can be designed as a result of the evidence acquired from the feasibility study.

This feasibility study was conducted to test the acceptability of incorporating an evidence-based TIPPS periodontal intervention into an ongoing maternal primary healthcare programme in a LMIC experiencing violence and conflict for pregnant women in their first trimester. This was an initial step to assess the methodological issues associated with proceeding to a full trial. In their paper, Bugge and Williams [13] suggested that as a result of examining the evidence from feasibility results, there are several possible outcomes which inform the conduct and design of a trial. These include (1) ‘adapting the intervention, (2) changing the context in which the intervention will be delivered, (3) amending the trial design, and (4) combination of [1]-[3].

The feasibility study provided evidence regarding the success of the recruitment, the retention, and acceptability and adherence with the TIPPS intervention. The women, antenatal nurses, and administrative staff appreciated the importance of the intervention and the requirement to provide toothbrushes, toothpastes, and interdental brushes to the participants. Therefore, the TIPPS intervention, in itself, did not require amendment; however, the context in terms of additional localities and the personnel to conduct the trial needed careful thought.

Recent research raised the importance of training midwives to identify pregnant women at risk of oral diseases, as they are the first contact many women have with healthcare [20]. Studies showed that midwives were able to recognise oral health care needs if they received a suitable training [21–24]. In this study, the situation was different. The antenatal nurses felt that, despite their TIPPS training, they did not have the specialist skills and knowledge nor the time to provide the TIPPS intervention. From the ADePT evaluation, it was apparent that the context in which the TIPPS intervention would be implemented required adjustment. The cost of providing dental health professionals and a dental clinic with all the necessary equipment to provide TIPPS, while supported by clinic staff, was felt to be financially prohibitive and unlikely in the current political situation. Moreover, since concerns regarding the time and costs of skilled nurses were voiced as unaffordable, an alternative solution needed to be considered. Community health care workers with their networks, who are closely working with their communities and clinics, would assist women to attend for antenatal care and permit the TIPPS intervention to be implemented in LMIC experiencing violence and conflict. While the majority of women attended the TIPPS appointments especially when they coincided with their antenatal appointments, three women were unable to attend since they were ‘under fire’ during the time of their appointments at Time 2. Therefore, since a full trial will be conducted in a conflict zone with fragile communities, it is possible that factors out-with the conduct of the trial may have a bearing on success. Thus, the necessity to increase the duration of the trial needs to be acknowledged and incorporated into the trial design. The feasibility study took place in a primary healthcare clinic situation in an urban area only. The process evaluation pointed to the importance of including other localities, such as rural and additional urban clinics, in a full trial. Therefore, the trial design would require amendment as a multicentred trial.

Due to the limited funds in the present study, the cost of providing periodontal and/or dental treatments has not been included. However, participating mothers and staff in the Primary Health Care in Palestine strongly recommended providing dental treatments as part of the intervention. Thus, this recommendation will be considered for the future RCT proposal.

It would have been useful to assess the costs for clinical staff’s time and use of clinical facilities, including the cost of providing the TIPPS intervention and periodontal and/or dental treatments. This was not possible in the feasibility study, although total costs of providing the materials for the intervention were known. Therefore, as a part of a full trial, an economic assessment, including cost efficiency and the costs of using community health workers should be incorporated into the trial design.

The suitability of the outcome measures was satisfactory and would be included and used in a full trial. An improvement in the gingival health of the pregnant women was shown, with infant birth weight being between 2.50 and 3.4 kg. Learning from this work, it would have been beneficial to collect a comparison treatment as a usual group to assess gingival health and infant birth weight as part of the feasibility study. This was not possible due to funding restrictions. Nevertheless, as a consequence of the feasibility work study, there is a need to reconsider the inclusion criteria regarding singleton/multiple births and to amend the trial design to stratify for first or previous pregnancies.

Studies in Gaza Strip revealed that smoking among females ranged from 1.2 to 27.1% [25–27]. Despite the fact that smoking is known to be one of the main periodontal and infant low birth weight risk factors, the current study was not able to measure the impact of this factor on periodontal health and infant birth weight. As previous research indicated that the women in Gaza Strip traditionally cannot reveal this information because of conservative cultural values which strictly forbids smoking among women [25]. However, the future randomised controlled trial should investigate the maternal smoking and the exposure to secondhand smoking in home or workplace.”

## Conclusion

Intervention to promote maternal oral health in Palestine and Middle East and North Africa regions are rare [28]. This feasibility study showed that it was possible to introduce and implement a new intervention into an established primary healthcare clinic for women and their children in areas of conflict and violence. It also showed that it was possible to collect appropriate outcome measures and that the TIPPS intervention improved maternal gingival health and improved infant birth weight. Therefore, the findings of this evaluation of the feasibility study demonstrated that an assessment of the methodological issues suggested progression to a full trial. The decision to change the context in which the TIPPS intervention would be delivered and the need to amend the proposed trial design was made as a result of the evidence acquired from the feasibility study. Therefore, the full trial would be multicentred, the inclusion criteria would be altered to include singleton births only, the analysis would be stratified by the number of previous pregnancies, the people providing the TIPPS intervention would be community health workers, and an economic evaluation would be included as an integral part of the trial design.

## Data Availability

Not applicable

## Abbreviations

TIPPS: Talk, Instruct, Practise and Plan and Support
LMIC: Low- and middle-income countries
ADePT: A process for Decision-making after Pilot and feasibility Trials
WHO: World Health Organisation
SPSS: Statistical Package for the Social Sciences
